# Preventive Effects of *Salacia reticulata* on Obesity and Metabolic Disorders in TSOD Mice

**DOI:** 10.1093/ecam/nep052

**Published:** 2011-06-23

**Authors:** Tomoko Akase, Tsutomu Shimada, Yukiko Harasawa, Tomohide Akase, Yukinobu Ikeya, Eiichi Nagai, Seiichi Iizuka, Gojiro Nakagami, Shinji Iizaka, Hiromi Sanada, Masaki Aburada

**Affiliations:** ^1^Graduate School of Medicine, The University of Tokyo, 7-3-1 Hongo, Bunkyo-ku, Tokyo 113-0033, Japan; ^2^Research Institute of Pharmaceutical Sciences, Musashino University, 1-1-20 Shinmachi Nishitokyo-shi, Tokyo 202-8585, Japan; ^3^Graduate School of Natural Science and Technology, Kanazawa University, Kakuma-machi Kanazawa-shi, Ishikawa 920-1192, Japan; ^4^Department of Pharmacy, Saiseikai Yokohama Tobu-Hospital, 3-6-1 Simosueyoshi Turumi-ku Yokohama-city, Kanagawa 230-0012, Japan; ^5^Department of Pharmacy, Ritsumeikan University, 1-1-1 Noji-higashi, Kusatsu, Shiga 525-8577, Japan; ^6^Graduate School of Medicine, Hokkaido University, North-15, West-7, Kita-ku, Hokkaido, 060-8638, Japan

## Abstract

The extracts of *Salacia reticulata* (*Salacia* extract), a plant that has been used for the treatment of early diabetes, rheumatism and gonorrhea in Ayurveda, have been shown to have an anti-obesity effect and suppress hyperglycemia. In this study, the effects of *Salacia* extract on various symptoms of metabolic disorder were investigated and compared using these TSOD mice and non-obese TSNO mice. Body weight, food intake, plasma biochemistry, visceral and subcutaneous fat (X-ray and CT), glucose tolerance, blood pressure and pain tolerance were measured, and histopathological examination of the liver was carried out. A significant dose-dependent decline in the gain in body weight, accumulation of visceral and subcutaneous fat and an improvement of abnormal glucose tolerance, hypertension and peripheral neuropathy were noticed in TSOD mice. In addition, hepatocellular swelling, fatty degeneration of hepatocytes, inflammatory cell infiltration and single-cell necrosis were observed on histopathological examination of the liver in TSOD mice. *Salacia* extract markedly improved these symptoms upon treatment. Based on the above results, it is concluded that *Salacia* extract has remarkable potential to prevent obesity and associated metabolic disorders including the development of metabolic syndrome.

## 1. Introduction

Ayurvedic medicine is an ancient system of healthcare that is native to the Indian subcontinent. It is presently in daily use by millions of people in India, Nepal and Sri Lanka [1]. *Salacia reticulata* is a climbing plant in the Hippocrateaceae family that has been used traditionally in Indian Ayurvedic medicine and is said to be effective for the prevention and treatment of diabetes, rheumatism, gonorrhea and skin diseases [2]. The major phytochemical components of *Salacia* include Triterpenes [3] such as antileukemic Isoiguesterin [4], Aldose reductase and *α*-glucosidase inhibitors, such as kotalanin 16-acetate [5], Kotalanol [2] and potent antioxidant, quinine methides [6], polyphenol constituents with *α*-glucosidase and aldose reductase inhibitory activities, Mangiferin [7, 8] and potent *α*-glucosidase inhibitor, Salacinol [9, 10]. There are evidences to suggest that the member of Salacia family are efficacious for treatment of metabolic disorders, obesity [11], hyperinsulinemia [12], cardiac lipid metabolism [13] and cardiac hypertrophy [14], and it is expected that the salacia family will be efficacious in the treatment of metabolic disorders. In contrast, Ratnasooriya et al. reported that a large dose of *S. reticulata* root extract could be hazardous to successful pregnancy in women [15].

In this study, we have investigated the preventive effects of *S. reticulata* on various disease states (diabetes, hyperlipidemia, hypertension and diabetic peripheral neuropathy) that accompany metabolic disorders. We focused on the visceral fat accumulation using a model animal, TSOD (Tsumura, Suzuki, Obese, Diabetes) mice, which are known to develop disease states similar to human metabolic disorders including visceral fat accumulation, glucose intolerance, hyperlipidemia, hypertension, hyperinsulinemia and peripheral neuropathy [16].

## 2. Methods

Male TSOD mice [16–21] and male TSNO (Tsumura Suzuki Non-Obese) mice were used (Institute of Animal Reproduction, Ibaragi). TSNO mice, produced from the same ancestors as TSOD mice, do not develop symptoms such as visceral fat accumulation, insulin resistance, glucose and lipid metabolism disorders and hypertension and were used as control animals [16–21]. TSOD and TSNO mice were purchased at the age of 3 weeks, and the experiments were started using 4-week-old mice, after 1 week of acclimation.

All animals were handled in accordance with the guidelines of the NRC, and this study was approved by the experimental animal ethics committee in Musashino University.

**Figure 1:**
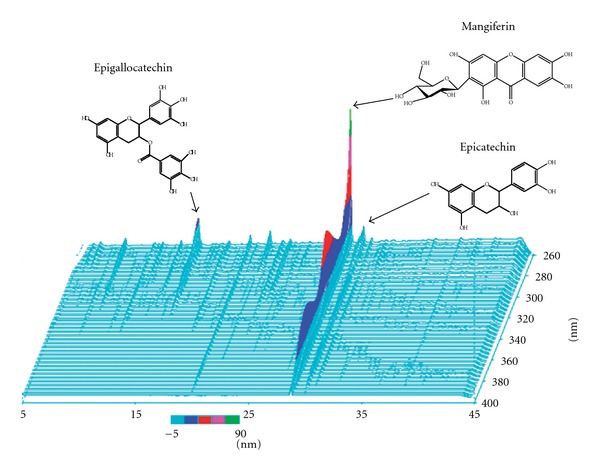
Analysis by three-dimensional HPLC of major chemical compounds in *Salacia reticulata*.

An extract of *S. reticulata* (available as extract powder. Kothalahim Japan Co., Ltd, Tokyo), identified by Srilanka Indigenous Medicine Exporters & Manufacturers Association, was further treated by adding branched cyclodextrin (Isoelite) to a hot-water suspension of the extract. The solvent was evaporated at low than 40°C and the remaining solution was spray-dried to obtain *Salacia* extract. The study feed was prepared by mixing the powder feed MF (Oriental Yeast Co., Ltd., Chiba, Japan) with *Salacia* extract at a concentration of 1.0% or 3.0%. The three-dimensional high-performance liquid chromatography (HPLC) chromatogram of the extract powder is shown in Figure 1. The polyphenol content, determined by the colorimetric method (Folin-Ciocalteu method), was 7.9%. The mangiferin content was 0.6% as determined by HPLC, using a Wakosil-II5C18AR column (Mobile phase: methanol and 1% acetic acid (10 : 90) as the mobile phase, detection at a wavelength of 360 nm, a column temperature of 35°C and a flow rate of 1 mL/min) (Wako Pure Chemical Industries, Ltd., Osaka, Japan).

### 2.1. Mode of Administration

After acclimation (1 week), TSOD and TSNO mice were divided into three groups so that the mean body weight of each group was uniform. Animals were allocated into one of the following groups:

1. TSOD mice groups:
  - Powder-fed MF group (TSOD control group),
  - 1.0% *Salacia* extract powder-treatment group (1% SR-TSOD group), and
  - 3.0% *Salacia* extract powder-treatment group (3% SR-TSOD group).
2. TSNO mice groups:
  - Powder-fed MF group (TSNO control group),
  - 1.0% *Salacia* extract powder-treatment group (1% SR-TSNO group), and
  - 3.0% *Salacia* extract powder-treatment group (3% SR-TSNO group).

Control groups were given *ad libitum* powder-feed MF from a feeder for 8 weeks.

### 2.2. Food Intake

During the experiment, animals were given tap water *ad libitum* for drinking, and were kept at a temperature of 23  ±  1°C and a relative humidity of 55  ±  5%, with a 12 h light-dark cycle with light time from 8:00 to 20:00. Animals were kept for 2 months. Food intake was determined every 2 weeks from the start of the experiment (4-week-old mice) by deducting the total weight of any remaining feed and the amount of spilled feed in each cage from the weight of the feeder of each cage filled with a constant amount of feed and then dividing each difference by the numbers of days and the number of animals to calculate a mean daily food intake per animal.

### 2.3. Changes in Body Weight and Visceral and Subcutaneous Fat

Body weight was measured every week during the period from the start of the study to the completion of the study after 8 weeks (12-week-old). At 4 and 8 weeks, mice were placed on an X-ray computed topography (CT) scanner for experimental animals, La Theta (ALOKA CO., LTD., Tokyo, Japan), under anesthesia with Nembutal (50 mg/kg, i.p.) and were scanned from the xiphisternum to sacrum at 1.5-mm intervals to determine visceral fat and subcutaneous fat.

### 2.4. Measurement of the Plasma Components

At 8 weeks, blood was drawn from the orbital venous plexus of the mice under nonfasting conditions without anesthesia, with a heparinized capillary tube. The collected blood was centrifuged and plasma was obtained to perform the examinations described below. Blood glucose level, total cholesterol level (T-Cho), triglyceride level (TG), glutamic-oxaloacetic transaminase (GOT) and glutamic-pyruvic transaminase (GPT) levels were determined by measuring absorbance using a Model 680 microplate reader (Bio-Rad, US), using the following biochemical test kits: Glucose CII-Test-Wako, Cholesterol E-Test-Wako, Triglyceride E-Test-Wako and Transaminase CII-Test-Wako (all kits used were made by Wako Pure Chemical Industries), respectively. In addition, blood was drawn from the caudal vena cava of the mice under ether anesthesia 8 weeks after start of the study, with a heparinized capillary tube, and plasma was obtained by centrifugation and used to determine insulin levels, using an insulin determination kit (ELISA method, Shibayagi Co., Ltd., Gunma, Japan) in a similar manner.

### 2.5. Glucose Tolerance Test

At 8 weeks, glucose was given orally (p.o) to the mice after an 18 h fast at a dose of 2 g/kg and blood glucose was determined immediately before administration of glucose (0 min) and 30, 60, 120 and 180 min after glucose administration. Blood samples were drawn from the orbital venous plexus, and plasma was obtained by centrifugation. The glucose level of each plasma sample was determined in a similar manner to that described in the previous section.

### 2.6. Blood Pressure

At 8 weeks, the blood pressure of each mouse was measured using a noninvasive blood pressure meter BP-98A (Softron, Co., Ltd, Tokyo, Japan). Each mouse was fixed in a positioner THC-2 (Softron, Co., Ltd) while body temperature was maintained at 37°C, and blood pressure at the base of the tail was determined by inserting the tail up to its base into the tail cuff.

### 2.7. Pain Test

The foot-pinch method as described by Suzuki et al. [17] was used to investigate response to pain sensation. At 8 weeks, the proximal part of the tarsus in the metatarsal region of the hind limb was pinched with an artery clip (BHO20R; pressure 300 g: Bulldog Clamp, Johns Hopkins, Tokyo, Japan), and the time until the mouse reacted and bit the clip on the hind limb was determined as a latent reaction time until the mouse felt pain. The mean latent reaction time of both hind limbs was regarded as the latent reaction time of each individual animal.

### 2.8. Histopathological Examination of the Liver

At 8 weeks, the mice were dissected to collect livers after drawing blood from the inferior vena cava of 12-week-old mice under ether anesthesia. After formalin fixation of collected livers, Hematoxylin and Eosin (HE)-stained sections were prepared and observed under an optical microscope (×40).

### 2.9. Statistical Analysis

The significant differences were evaluated using Dunnett's multiple comparison test.

## 3. Results

### 3.1. Food Intake

No differences in food intake were observed between TSOD mice (4.5  ±  0.1 g/1 mouse/day (5W)) and TSNO mice [4.0  ±  1.3 g/1 mouse/day (5W)].

### 3.2. Changes in Body Weight and Visceral and Subcutaneous Fat

#### 3.2.1. Growth Curve

The TSOD control group showed significant body weight gain compared with the TSNO control group throughout the entire experimental period, from the start to the completion of the study (Table 1). In both TSOD and TSNO mice, *Salacia* extract-treatment groups showed significant and dose-dependent decreases in the body weight within 1 week after the start of treatment, and the 1% and 3% TSOD groups, in particular, showed marked decreases in body weight.

#### 3.2.2. Changes in Visceral and Subcutaneous Fat

**Figure 2:**
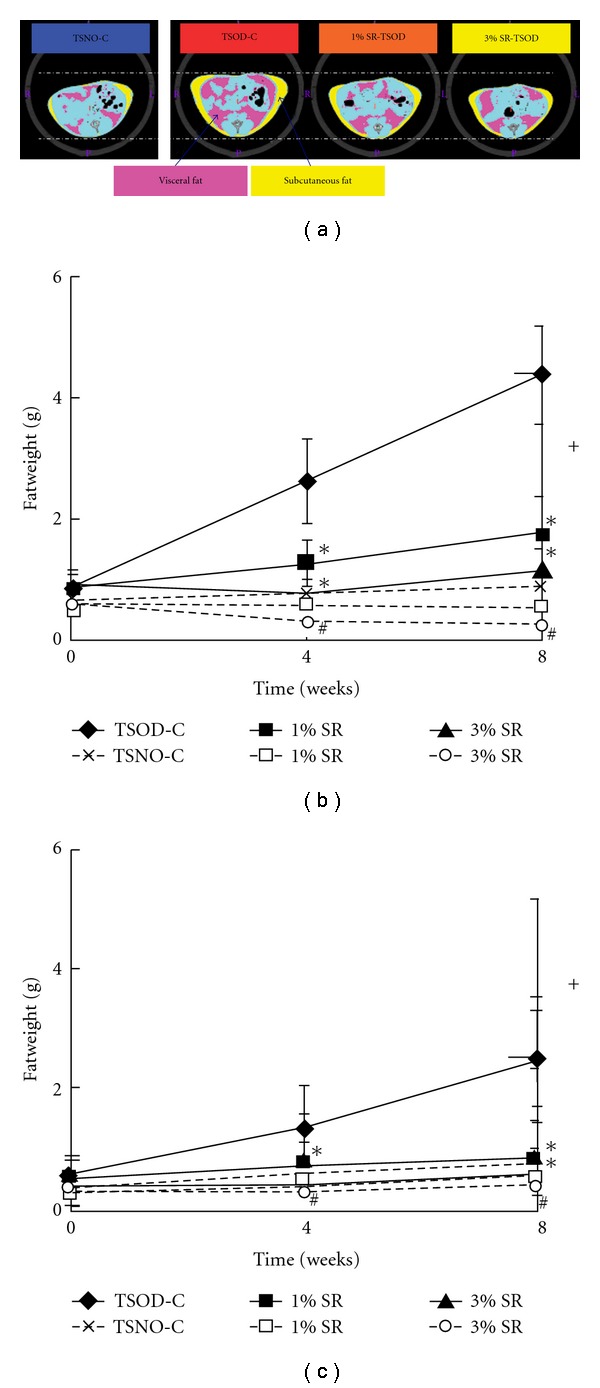
The effect of *Salacia reticulata* on the accumulation of adipose tissue in the TSOD and TSNO mice. (a) Computed topography scanner. (b) Visceral fat. (c) Subcutaneous fat. Data represent the mean  ±  SD of the results in six to nine animals. *P < .05 (versus TSOD control); ^#^ *P* < .05 (versus TSNO control); ^+^ *P* < .05 (TSOD control versus TSNO control); pink zone: visceral fat; yellow zone: subcutaneous fat.

Figure 2 shows the amount of both visceral and subcutaneous fat in the TSOD control group increased significantly compared with the TSNO control group at 4 and 8 weeks after start of the study, and the differences increased with time. On the other hand, among the TSOD mice, the treatment groups showed significant, dose-dependent decrease in both visceral and subcutaneous fat compared with the TSOD control group, and the differences increased with time. Among TSNO mice, the treatment groups also showed dose-dependent decrease in the amount of both visceral and subcutaneous fat compared with the control group and the difference significant in the 3% *Salacia* extract-treated group.

### 3.3. Measurement of Plasma Components

**Figure 3:**
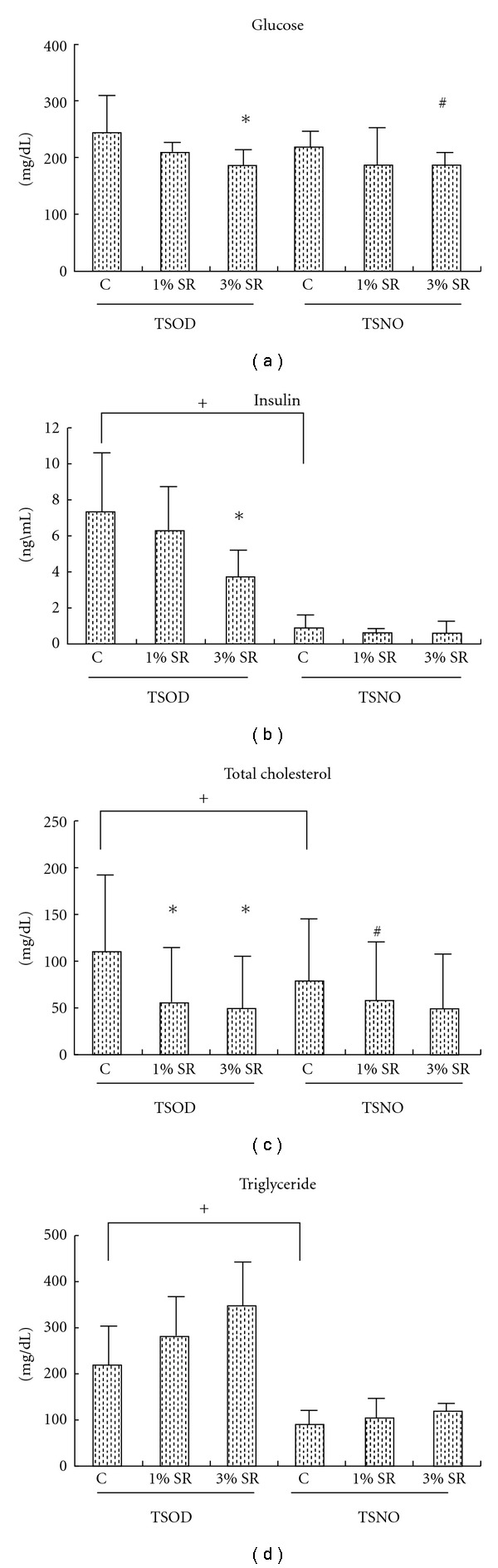
The effect of *Salacia reticulata* on the biochemical parameters of plasma in the TSOD and TSNO mice at 8 weeks. Data represent the mean  ±  SD of the results in six to nine animals. *P < .05 (versus TSOD control); ^#^ *P* < .05 (versus TSNO control); ^+^ *P* < .05 (TSOD control versus TSNO control).

The TSOD control group showed significantly higher levels of insulin, T-Cho and TG than the TSNO control group, but no significant differences in blood glucose were observed between the control groups (Figure 3). Between-group comparisons in the TSOD cohort, the 1% and 3% treated group showed dose-dependent decrease in levels of blood glucose, insulin and T-Cho, compared to control group. The decrease in the levels of blood glucose and insulin were significant for 1% treated-TSOD group. Similarly, among the TSNO cohort, the 1% group showed a significant decrease in T-Cho, while the 3% group showed a significant decrease in blood glucose level.

### 3.4. Glucose Tolerance Test

**Figure 4:**
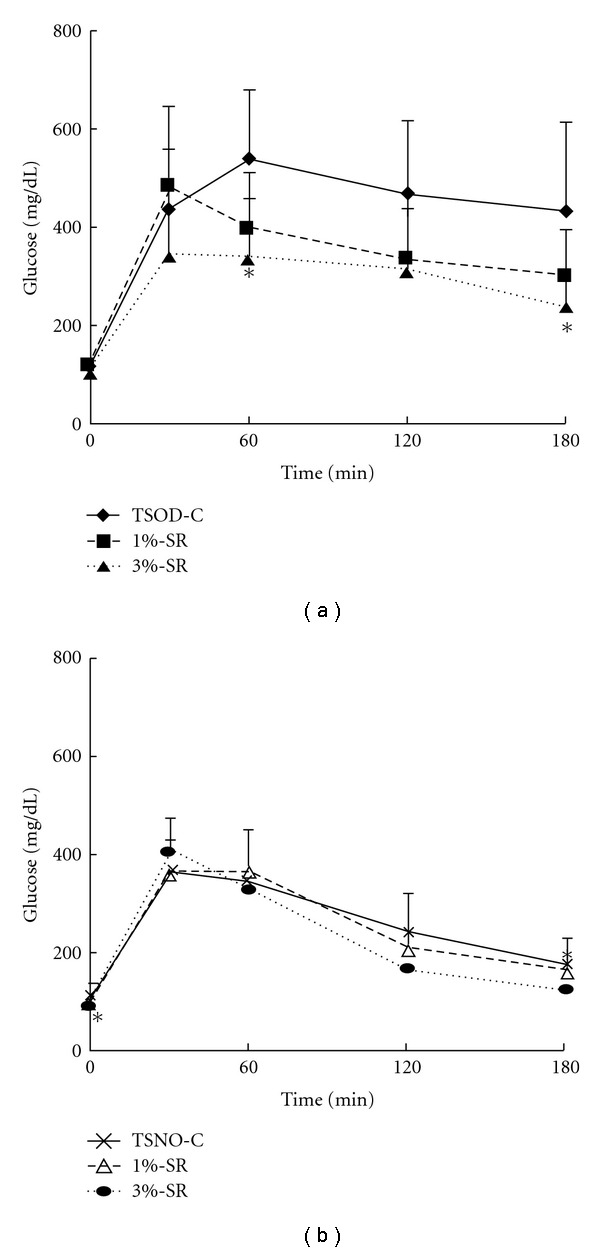
Changes of the plasma glucose levels in the oral glucose test. Data represent the mean  ±  SD of the results in six to nine animals. *P < .05 (versus TSOD control); ^#^ *P* < .05 (versus TSNO control). Significant differences were noted between the TSOD control mice and TSNO control mice at 120 and 180 min after the start of the oral glucose tolerance test.

The TSOD control group showed significantly higher blood glucose levels from 60 to 180 min after glucose loading, compared with the time course of changes in the blood glucose levels in the TSNO control group, with significant differences apparent at 120 and 180 min after glucose loading, indicating a reduction in glucose tolerance in the TSOD mice (Figure 4). Among TSOD mice, both the 1%- and the 3%-treated groups showed significant decrease in blood glucose levels compared to the control group from 60 to 180 min after glucose loading. Similarly, the 3%-treated TSNO group showed a significant decrease in blood glucose compared relative to the TSNO control group at 180 min.

### 3.5. Blood Pressure

The TSOD control group showed significant elevations in SBP, DBP and MBP values, compared with the TSNO control group (Table 2). In TSOD mice, both the 1%- and 3%-treated groups showed dose-dependent suppression of SBP, DBP and MBP values, compared to control group. The antihypertensive effect was significant in each dose group. In the TSNO mice, only the 3%-treated TSNO group showed significant suppression of SBP, DBP and MBP values, compared to control group.

### 3.6. Pain Test

The TSOD control group showed significantly longer latent reaction time than the TSNO control group (Table 3). Regarding this prolonged latent reaction time in the TSOD control group, the 1% and 3%-treated groups both showed significant, dose-dependent reductions in latent reaction time. In contrast, there was little between-group difference in TSNO mice in latent reaction time.

### 3.7. Histopathological Examination of the Liver

**Figure 5:**
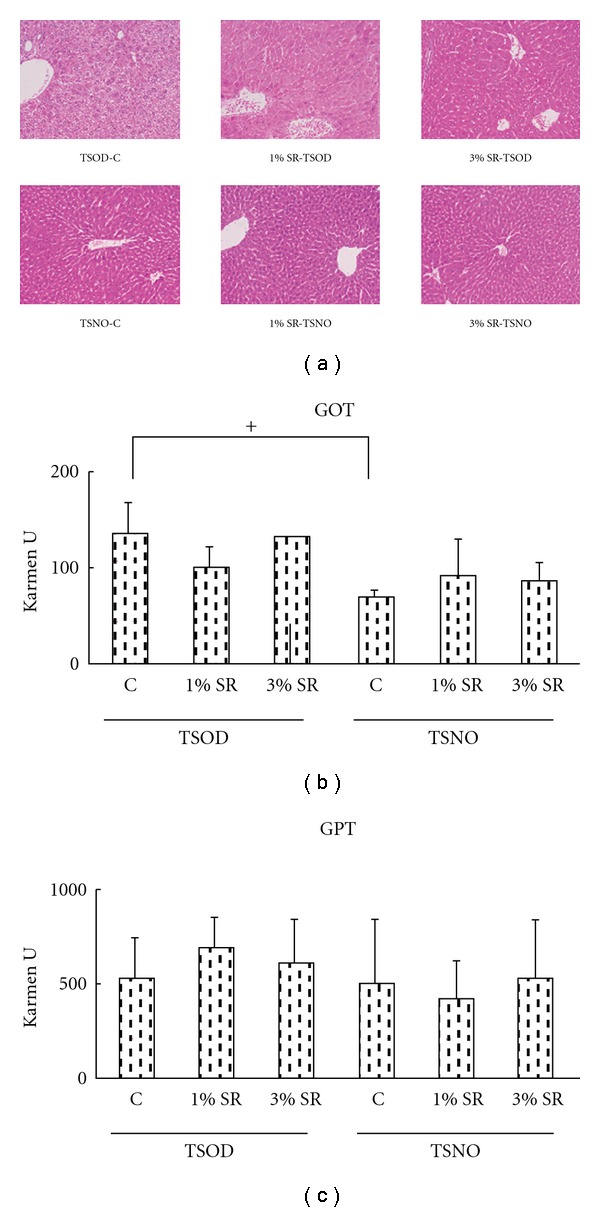
Effect of *Salacia reticulata* on liver histology (×40) and liver function test. GOT denotes glutamate oxaloacetate transaminase; GPT denotes glutamate pyruvate transaminase.

The TSOD control group showed marked enlargement of hepatocytes and fatty degeneration, due to accumulation of fine lipid droplets, compared to the TSNO control group (Figure 5). In addition, infiltration of inflammatory cells and single-cell necrosis were observed in the TSOD control group. Compared to TSOD control group, enlargement of hepatocytes was suppressed in a dose-dependent manner in the 1% and 3%-treated groups, and fatty degeneration of hepatocytes, infiltration of inflammatory cells and single-cell necrosis were also improved. In contrast, none of the TSNO mice groups showed any changes in the liver. Among the liver function test items, Salacia extract treatment did not affect GOT and GPT in either TSOD or TSNO mice (Figure 5).

## 4. Discussion

In this study, we compared the preventive effects *Salacia* extract on metabolic disorders, including obesity using TSOD mice, a model of obesity with that of TSNO mice that had not developed obesity and/or various symptoms of metabolic syndrome (MS). Increase in body weight was found to be significantly higher in the TSOD groups than in the TSNO groups, from the early stage of the experiment. The TSOD mice became markedly obese. In both TSOD and TSNO mice, *Salacia* extract treatment was associated with dose-dependent decrease in body weight with time. At 8 weeks after initiation of treatment, body weight gain was suppressed by approximately 36% in the 3%-treated TSOD group compared to control, while body weight gain was also suppressed in the TSNO groups, showing stronger suppression of body weight gain in TSOD obese mice. Since there were no differences in food intake between the groups and the liver function tests showed no abnormal GOT and GPT, it was suggested that the suppression of body weight gain due to treatment was not caused by suppression of food intake or a toxic effect.

It is thought that obesity is mainly caused by fat accumulation. The TSOD control group showed significantly greater visceral and subcutaneous fat accumulation than the TSNO control group, and the accumulation in each group mostly paralleled the time course of body weight gain. *Salacia* extract markedly suppressed both visceral and subcutaneous fat accumulation in the TSOD mice and the suppression was most notable when comparing the 3%-treated TSOD group and the control group. It was suggested that the suppression of fat accumulation by *Salacia* extract was a factor in the suppression of body weight gain. Furthermore, *Salacia* extract also significantly suppressed both visceral and subcutaneous fat accumulation in the 3% TSNO group, compared to the control group, showing similar results for body weight gain. Yoshikawara et al. reported the anti-obesity effect of *Salacia reticulata* in Zucker fatty rats and SD rats fed a high-fat diet [11]. Koshino et al. examined the effect of *Salacia* extract on the mice fed a high-fat diet, and reported that *Salacia* extract suppressed visceral fat accumulation [22]. This study also showed that *Salacia* extract suppressed body weight gain, but it was suggested that the suppression of visceral and subcutaneous fat accumulation largely contributed to the anti-obesity effect.

Since visceral fat accumulation is known to be a fundamental cause of various symptoms of MS, it is expected that the suppression of visceral fat accumulation by *Salacia* extract would prevent various disease states following insulin resistance, and therefore the following investigation was performed.

It is known that insulin resistance resulting from visceral fat accumulation is involved in various metabolic disorders. Compared to the TSNO control group, hyperinsulinemia and abnormal glucose tolerance were observed in the TSOD control group, suggesting that TSOD mice developed insulin resistance. In the TSOD mice, *Salacia* extract treatment decreased blood glucose levels and insulin levels, and improved abnormal glucose tolerance in a dose-dependent manner. In contrast, in the TSNO mice, the 3%-treated group showed decreases in blood glucose levels and partially significant decreases in the glucose tolerance. It has been reported that salacinol and kotalanol contained in *Salacia* have an *α*-glucosidase inhibitory effect comparable to acarbose, which is used clinically for the inhibition of postprandial hyperglycemia [8, 10], and it has been found that 13-membered ring thiocyclitol, which was detected in *Salacia*, also has a potent *α*-glucosidase inhibitory effect [23, 24]. It was suggested that decreased absorption of glucose derived from dietary carbohydrates due to this *α*-glucosidase inhibitory effect of *Salacia* may be involved in the blood glucose-lowering effects observed in both TSOD and TSNO mice groups in this study. It has also been suggested that the suppressive effect of *Salacia* extract on visceral fat accumulation contributed to the improved insulin resistance in the TSOD mice groups. It has been reported that *S*. *reticulata* improved hemoglobin A1c in patients with type 2 diabetes [25] and suppressed increases in blood glucose and blood insulin levels [26] in clinical studies, and it is assumed that *S*. *reticulata* improves glucose metabolism.

The TSOD control group showed significant increases in plasma T-Cho and TG, compared with the TSNO control group, and the TSOD control group showed symptoms of abnormal lipid metabolism. On histopathological examination of the liver, fatty degeneration was observed in the TSOD control group. *Salacia* extract significantly lowered T-Cho levels in a dose-dependent manner and improved liver degeneration in the TSOD mice. At the same time, *Salacia* extract also lowered blood T-Cho levels in the TSNO mice groups. Both TSOD and TSNO mice groups showed no significant differences in blood TG levels due to treatment with *Salacia* extract. *Salacia* extract is known to increase the activity of hormone-sensitive lipase involved in lipolysis [26], and Huang et al. [27, 28] reported that treatment with S. *oblonga*, a plant that is a member of the same species as *S*. *reticulata*, induced expression of peroxisome proliferator-activated receptor (PPAR)-*α* regulatory genes and protein expression (PPAR*α*, CPT1, ACO), which are involved in *β*-oxidation in the liver in Zucker diabetes rats. In addition, it has been reported that polyphenolic constituents contained in *Salacia* extract exhibit a lipolytic action by inhibiting enzymes involved in lipid metabolism, such as pancreatic lipase, lipoprotein lipase and glucose-6-phosphate ester dehydrogenase [11]. On the basis of the above findings, this study suggests that *Salacia* extract decreases fatty weight and improves degeneration of hepatocytes by acting to facilitate lipolysis in the liver and adipocytes. In contrast, it has been suggested that plasma TG levels are not improved as a result of transfer of free fatty acids produced by lipolysis from liver tissues and adipose cells to plasma. Since it is known that accumulation of adipose tissues and fat in the liver evoke insulin resistance, it was suggested that the suppression of fat accumulation and reduction in hepatic lipids due to *Salacia* extract treatment partially contributed to improved insulin resistance.

On histopathological examination of the liver, gross swelling of the liver was seen in the TSOD control group, and the liver was yellowish-white in color. Histologically, extensive fatty degeneration of hepatocytes due to accumulation of fine lipid droplets was found, and it was considered that the liver had progressed to fatty liver. These changes are similar to those found in the livers of patients with Nonalcoholic Fatty Liver Disease (NAFLD) and were considered to be affected by diabetes. It is known that human NAFLD progresses to nonalcoholic steatohepatitis (NASH) by second hit of adipocytokines and inflammatory cytokines [29]. In the TSOD mice groups, infiltration of inflammatory cells including neutrophils, single-cell necrosis and hepatocellular swelling were also observed. In addition, when feeding TSOD mice without treatment for a long time, they develop liver tumors at high rates. Based on these findings, it was considered that livers of the TSOD control group appeared similar to that of livers in humans with NASH, and had progressed from simple fatty liver to the initial disease state in NASH. *Salacia* extract suppressed these changes and it has been inferred that the suppression was mainly caused by decreased body fat and improvement of diabetes. While it cannot be concluded definitively that *Salacia* extract suppresses the development of liver tumors in the TSOD control group, *Salacia* extract may improve human NASH.

The effect on hypertension, one of the symptoms of MS, was examined. SBP, DBP and MBP levels in the 12-week-old TSOD control group at the completion of the experiment were significantly higher than those in the TSNO control group, indicating that the TSOD control group was hypertensive. *Salacia* extract significantly decreased the blood pressure in TSOD mice in a dose-dependent manner, indicating that *Salacia* extract has a preventive effect on hypertension, one of the symptoms of MS. Moreover, *Salacia* extract also showed a significant hypotensive effect in the 3% *Salacia* extract TSNO group. Hung et al. reported that *S*. *oblonga* root extract suppressed angiotensin II-stimulated hypertrophic responses and protein synthesis in heart-derived H9c2 cells and angiotensin II-accelerated hyperplasia in rat cardiac fibroblasts [30]. Hung et al. [31] also reported that *S*. *oblonga* decreased cardiac AT1 receptors. Since angiotensin II elevates blood pressure, it was suggested that angiotensin II was involved in the decreases in blood pressure due to *Salacia* extract treatment observed in this experiment. Additionally, visceral fat accumulation may contribute to the development of hypertension in TSOD mice, and it was also suggested that the suppression of visceral fat accumulation was involved in the *Salacia* extract-treatment-associated decrease in blood pressure.

We have already reported that peripheral neuropathy develops as a diabetic complication in TSOD mice [16, 31–33]. The latent reaction time was significantly longer in the TSOD control group than in the TSNO control group, and it was suggested that peripheral neuropathy had developed in the TSOD control group. In addition, *Salacia* extract treatment significantly decreased latent reaction time in the TSOD mice groups in a dose-dependent manner. It is known that advanced glycation end products (AGEs) are produced in diabetic hyperglycemia and that these AGEs contribute to diabetic peripheral neuropathy. It was suggested that the hypoglycemic effect of *Salacia* extract shown in this study prevented peripheral neuropathy by suppressing AGE production.

In conclusion, it may be stated that *Salacia* extract treatment decreases body weight, visceral and subcutaneous fat accumulation and blood glucose, improvement of abnormal lipid metabolism, fatty liver and enlarged liver. A decrease in blood pressure and improvement of peripheral neuropathy in TSOD mice suggested that *Salacia* extract has the potential to prevent the development of the major disease states seen in MS. Furthermore, *Salacia* extract treatment was associated with suppression of body weight gain, decreases in plasma T-Cho levels and decreased blood pressure in TSNO mice, the control mice, suggesting that *Salacia* extract treatment at the level used in this study has utility in healthy subjects.

## Funding

“High-Tech Research Center” Project for Private University: matching fund subsidy from MEXT (Ministry of Education Culture, Sports, Science and Technology) 2004–08 of Japan.

## Figures and Tables

**Table 1:** The effect of *Salacia reticulata* on the body weight of the TSOD and TSNO mice.

	TSOD-C	1% SR-TSOD	3% SR-TSOD	TSNO-C	1% SR-TSNO	3% SR-TSNO
0 week	22.2 ± 3.2	21.7 ± 2.9	21.8 ± 3.0	12.9 ± 1.4	12.6 ± 1.1	12.7 ± 1.2
4 weeks	39.7 ± 4.1*	34.1 ± 3.9*	28.1 ± 4.3*	31.2 ± 1.4^#^	28.9 ± 1.6^#^	26.1 ± 0.9^#^
8 weeks	47.4 ± 4.5*	38.0 ± 2.8*	30.6 ± 3.8*	34.3 ± 1.6^#^	31.0 ± 1.8^#^	30.1 ± 0.6^#^

Data represent the mean  ±  SD of the results in 6–9 animals.

*P < .05 (versus TSOD control); ^#^ *P* < .05 (versus TSNO control).

**Table 2:** The effect of *Salacia reticulata* on the blood pressure of the TSOD and TSNO mice at 8 weeks.

	TSOD-C	1% SR-TSOD	3% SR-TSOD	TSNO-C	1% SR-TSNO	3% SR-TSNO
SBP	107.6 ± 6.7^+^	94.1 ± 5.6*	90.5 ± 8.7*	94.1 ± 5.6^+^	93.8 ± 9.5	86.8 ± 9.6^#^
DBP	74.4 ± 7.3^+^	67.0 ± 5.3*	54.7 ± 11.4*	67.0 ± 5.3^+^	63.3 ± 7.6	55.5 ± 8.5^#^
MBP	85.4 ± 5.7^+^	75.9 ± 5.1*	66.5 ± 10.0*	75.9 ± 5.1^+^	73.3 ± 7.7	65.9 ± 8.2^#^

Data represent the mean  ±  SD of the results in 6–9 animals.

*P < .05 (versus TSOD control); ^#^ *P* < .05 (versus TSNO control); ^+^ *P* < .05 (TSOD control versus TSNO control); SBP, systolic blood pressure; DBP, diastoric blood pressure; MBP, mean blood pressure.

**Table 3:** The effect of *Salacia reticulata* on the peripheral neuropathy of the TSOD and TSNO mice at 8 weeks.

TSOD-C	1% SR-TSOD	3% SR-TSOD	TSNO-C	1% SR-TSNO	3% SR-TSNO
7.5 ± 2.7^+^	4.5 ± 2.3*	2.9 ± 0.4*	4.8 ± 2.7^+^	4.4 ± 1.7	5.3 ± 1.3

Data represent the mean  ±  SD of the results in 6–9 animals.

*P < .05 (versus TSOD control); ^#^ *P* < .05 (versus TSNO control); ^+^ *P* < .05 (TSOD control versus TSNO control).

## References

[1] Cooper EL (2008). Ayurveda is embraced by eCAM. *Evidence-Based Complementary and Alternative Medicine*.

[2] Yoshikawa M, Murakami T, Yashiro K, Matsuda H (1998). Kotalanol, a potent alpha-glucosidase inhibitor with thiosugar sulfonium sulfate structure, from antidiabetic ayurvedic medicine Salacia reticulata. *Chemical & Pharmaceutical Bulletin*.

[3] Tewari NC, Ayengar KNN, Rangaswami S (1974). Triterpenes of the root-bark of Salacia prenoides DC. *Journal of the Chemical Society*.

[4] Sneden AT (1981). Isoiguesterin, a new antileukemic bisnortriterpene from Salacia madagascariensis. *Journal of Natural Products*.

[5] Matsuda H, Murakami T, Yashiro K, Yamahara J, Yoshikawa M (1999). Antidiabetic principles of natural medicines. IV. Aldose reductase and qlpha-glucosidase inhibitors from the roots of Salacia oblonga Wall. (Celastraceae): structure of a new friedelane-type triterpene, kotalagenin 16-acetate. *Chemical & Pharmaceutical Bulletin*.

[6] Figueiredo JN, Räz B, Séquin U (1998). Novel quinone methides from Salacia kraussii with in vitro antimalarial activity. *Journal of Natural Products*.

[7] Karunanayake EH, Sirimanne SR (1985). Mangiferin from the root bark of Salacia reticulata. *Journal of Ethnopharmacology*.

[8] Yoshikawa M, Nishida N, Shimoda H, Takada M, Kawahara Y, Matsuda H (2001). Polyphenol constituents from Salacia species: quantitative analysis of mangiferin with alpha-glucosidase and aldose reductase inhibitory activities. *Yakugaku Zasshi*.

[9] Ghavami A, Johnston BD, Pinto BM (2001). A new class of glycosidase inhibitor: synthesis of salacinol and its stereoisomerst. *Journal of Organic Chemistry*.

[10] Yoshikawa M, Morikawa T, Matsuda H, Tanabe G, Muraoka O (2002). Absolute stereostructure of potent *α*-glucosidase inhibitor, salacinol, with unique thiosugar sulfonium sulfate inner salt structure from Salacia reticulata. *Bioorganic and Medicinal Chemistry*.

[11] Yoshikawa M, Shimoda H, Nishida N, Takada M, Matsuda H (2002). Salacia reticulata and its polyphenolic constituents with lipase inhibitory and lipolytic activities have mild antiobesity effects in rats. *Journal of Nutrition*.

[12] Huang TH, Peng G, Li GQ, Yamahara J, Roufogalis BD, Li Y (2006). Salacia oblonga root improves postprandial hyperlipidemia and hepatic steatosis in Zucker diabetic fatty rats: activation of PPAR-*α*. *Toxicology and Applied Pharmacology*.

[13] Huang TH-W, Yang Q, Harada M (2006). Salacia oblonga root improves cardiac lipid metabolism in Zucker diabetic fatty rats: modulation of cardiac PPAR-*α*-mediated transcription of fatty acid metabolic genes. *Toxicology and Applied Pharmacology*.

[14] Huang TH, He L, Qin Q (2008). Salacia oblonga root decreases cardiac hypertrophy in Zucker diabetic fatty rats: inhibition of cardiac expression of angiotensin II type 1 receptor. *Diabetes, Obesity and Metabolism*.

[15] Ratnasooriya WD, Jayakody JR, Premakumara GA (2003). Adverse pregnancy outcome in rats following exposure to a Salacia reticulata (Celastraceae) root extract. *Brazilian Journal of Medical and Biological Research*.

[16] Iizuka S, Suzuki W, Tabuchi M (2005). Diabetic complications in a new animal model (TSOD mouse) of spontaneous NIDDM with obesity. *Experimental Animals*.

[17] Suzuki W (1998). TSOD mice. *Diabetes Frontier*.

[18] Suzuki W, Iizuka S, Tabuchi M (1999). A new mouse model of spontaneous diabetes derived from ddY strain. *Experimental Animals*.

[19] Suzuki W, Tabuchi M (1999). A new spontaneous type II diabetes model, TSOD mice (1): concerning the disease status. *Laboratory Animal Science and Technology*.

[20] Suzuki W, Iizuka S, Tabuchi M, Yanagisawa T, Kimura M (2002). A new spontaneous type II diabetes model. *Diabetes Frontier*.

[21] Suzuki W, Nakajima H (2005). Metabolic syndrome-related disease status of TSOD mouse. *Laboratory Animal Science and Technology*.

[22] Kishino E, Ito T, Fujita K, Kiuchi Y (2006). A mixture of the Salacia reticulata (Kotala himbutu) aqueous extract and cyclodextrin reduces the accumulation of visceral fat mass in mice and rats with high-fat diet-induced obesity. *Journal of Nutrition*.

[23] Ozaki S, Oe H, Kitamura S (2008). *α*-glucosidase inhibitor from Kothala-himbutu (Salacia reticulata WIGHT). *Journal of Natural Products*.

[24] Oe H, Ozaki S (2008). Hypoglycemic effect of 13-membered ring thiocyclitol, a novel *α*-glucosidase inhibitor from kothala-himbutu (Salacia reticulata). *Bioscience, Biotechnology and Biochemistry*.

[25] Kataoka K (2006). The hypoglycemic potential and safety profile of Salacia reticulate Wight extract powder in healthy and diabetic subjects. *New Diet Therapy*.

[26] Tanimura C, Terada I, Hiramatu K (2005). Effect of a mixture of aqueous extract from Salacia reticulate (Kotala himbutu) and cyclodextrin on the serum glucose and the insulin levels in sucrose tolerance test and on serum glucose level changes and gastro-intestinal disorder by massive ingestion. *Journal of the Yonago Medical Association*.

[27] Huang TH-W, Yang Q, Harada M (2006). Salacia oblonga root improves cardiac lipid metabolism in Zucker diabetic fatty rats: modulation of cardiac PPAR-*α*-mediated transcription of fatty acid metabolic genes. *Toxicology and Applied Pharmacology*.

[28] Huang TH, Peng G, Li GQ, Yamahara J, Roufogalis BD, Li Y (2006). Salacia oblonga root improves postprandial hyperlipidemia and hepatic steatosis in Zucker diabetic fatty rats: activation of PPAR-*α*. *Toxicology and Applied Pharmacology*.

[29] Day CP, James OFW (1998). Steatohepatitis: a tale of two “hits”?. *Gastroenterology*.

[30] Li Y, Huang TH-W, Yamahara J (2008). Salacia root, a unique Ayurvedic medicine, meets multiple targets in diabetes and obesity. *Life Sciences*.

[31] Tsunakawa M, Shimada T, Suzuki W (2006). Preventive effects of Daisaikoto on metabolic disorders in spontaneous obese type II diabetes mice. *Journal of Traditional Medicines*.

[32] Shimada T, Kudo T, Akase T, Aburada M (2008). Preventive effects of bofutsushosan on obesity and various metabolic disorders. *Biological and Pharmaceutical Bulletin*.

[33] Shimada T, Akase T, Kosugi M, Aburada M eCAM preventive effect of boiogito on metabolic disorders in the TSOD mouse, a model of spontaneous obese type II diabetes mellitus.

